# The Professional Union of Trained Nurses

**Published:** 1920-05-01

**Authors:** 


### THE PROFESSIONAL UNION OF TRAINED NURSES.

Under the auspices of this Union a "public meeting for nurses" was held on Saturday afternoon, the 24th inst., at St. George's Hall, in the Y.M.C.A. building, Tottenham Court Road. There was a fair attendance.

**Mrs. Winifred Paul** (the Chairman) said the meeting was called to celebrate their registration as a trade union. At the end of last year two meetings were held to discuss the advisability of forming a trade union for nurses, as a result of which they set to work, enrolling the first members on January 1. In the middle of March they were a duly registered trade union under the Acts. Since that date they had prospered, and had taken offices at Evelyn House, Oxford Street. In time they hoped to own a building, so that they could have their own lecture hall and club, employment bureau, and hostel—at all events for London. The Union was now paying its way, and new members were being enrolled. The Union wanted to see all nurses registered. A Scottish branch of the Union had been established in Glasgow, and it was growing. A public-health section of the Union was also being formed, as they felt that public-health workers had a grievance. Advertisements of vacancies for nurses were being watched, and if the salaries offered were not adequate the Union wrote protesting. Generally speaking, courteous replies had been received. Much fear had been expressed that if nurses joined the Union they would be called upon to strike; but she wished to make it quite clear that the Union had no strike clause in its constitution. They had a scheme for the sick members, whereby nurses could pay a little extra on the ordinary subscription, and beds were taken in the various hospitals in London, and when members were ill they had only to write or telephone to the Union and arrangements would be made for their treatment. A Hospital Sub-Committee was being formed in London to visit any member who was taken ill. She had heard of nurses ill in a room and trying to live on ten shillings a week.

**Dr Welply** (Medico-Political Union) conveyed to the meeting the congratulations of his Council on their becoming a trade union; also on the Secretary, Miss MacCallum, being a representative on the first Nursing Council. He regarded this Union as the most important of all the nursing organisations, because of the power it gave: he did not mean that nurses should not join other organisations. People would be sure to say it was derogatory to the nursing profession to have a trade union, and they would be asked if they contemplated striking against the sick and suffering. The weapon of the strike had been used in industrial spheres with great effect: he was not saying it had always been employed wisely. He had been sorry to hear Mrs. Paul say the Union of Nurses had no power to strike. He did not advocate striking against the sick and suffering, and there need be no fear that the medical profession would do that, but some day nurses would be employed by the State, and then they might need provision in the constitution for a strike in order to get reasonable demands granted. A revolver was much more efficacious when presented at anyone if it was known to be loaded.

**Miss Parsons** stated the case from the matron's point of view. She wished to tell nurses that they ought to be managing their own affairs. For a number of years nurses had been too lethargic about things; all their affairs—daily routine, off time—had been in the hands of other people; they had not been allowed to have a voice in what they would like, and, except rarely, they had not been allowed to ask for anything they wanted. If she had her way with hospitals and any institution run for the benefit of the sick and suffering and run by public funds, she would have a percentage of working members on the board of management representing every grade. She hoped for the day when every branch of hospital work would have no methods which would not bear the light of day. What could nurses do to bring about an improvement? Nurses individually could not do much, but they could if banded together. Another means was by co-operation.

**Mr. Naylor, J.P.,** said his connection with Trade Unionism had been a long one, and he argued that some such organisation was needed by nurses in order to remove their hardships. He was a Labour man, but he did not believe in extremes of any sort. In the early stages of their movement nurses must not be led aside by any will o' the wisps or anything of an idealistic character. In the aim at progress there would be no room for extreme policy; all possible should be done to get public opinion on their side. And he would specially say, "When you condemn do not be too sweeping."

**Miss Alderman** addressed herself to the Public Health aspect. She said the draft regulations for the training of health visitors required close examination by nurses and doctors. If the Ministry of Health had been in existence two or three years longer, a minimum of three years' hospital training would have been laid down, and the deficiency of numbers would have been made up by the release of well qualified and experienced sisters from military hospitals. If the nurses in the Public Health services had been properly organised, the unsatisfactory regulations could have been opposed.

**The Chairman** (Mrs. Paul) contended that the hospital was the institution which should train the nurse in preventive work; she should not have to go outside for that training, nor for midwifery. Most hospitals taught midwifery, but not to their nurses.

Questions were invited, and in reply to one concerning the strike clause, the Chairman said it might possibly be necessary at some future date to have such a clause. In the case of a grievance which was not remedied by the ordinary methods she preferred the boycott method. The intention of the Union was to obey the law.

**Miss MacCallum** advocated publicity as the great means for remedying abuses.

